# Epidermal *Nbn* deletion causes premature hair loss and a phenotype resembling psoriasiform dermatitis

**DOI:** 10.18632/oncotarget.8470

**Published:** 2016-03-30

**Authors:** Philipp Seidel, Martina Remus, Michael Delacher, Paulius Grigaravicius, David E. Reuss, Lucien Frappart, Andreas von Deimling, Markus Feuerer, Amir Abdollahi, Pierre-Olivier Frappart

**Affiliations:** ^1^ Molecular and Translational Radiation Oncology, National Center for Tumor Diseases (NCT), Heidelberg University Medical School (HUMS), Heidelberg, Germany; ^2^ German Cancer Consortium (DKTK) and Heidelberg Institute of Radiation Oncology (HIRO), German Cancer Research Center (DKFZ), Heidelberg, Germany; ^3^ Clinical Cooperation Unit Neuropathology, German Cancer Research Center (DKFZ), Heidelberg, Germany; ^4^ Helmholtz Young Investigator Group Immune Tolerance, Tumor Immunology Program, German Cancer Research Center, Heidelberg, Germany; ^5^ Department of Neuropathology, Institute of Pathology, Ruprecht-Karls-Universität Heidelberg, Heidelberg, Germany; ^6^ Leibniz Institute for Age Research - Fritz Lipmann Institute (FLI), Jena, Germany

**Keywords:** inflammation, Nbn, psoriasiform dermatitis, skin, Gerotarget

## Abstract

Nijmegen Breakage Syndrome is a disease caused by *NBN* mutations. Here, we report a novel function of Nbn in skin homeostasis. We found that Nbn deficiency in hair follicle (HF) progenitors promoted increased DNA damage signaling, stimulating *p16^Ink4a^* up-regulation, Trp53 stabilization and cytokines secretion leading to HF-growth arrest and hair loss. At later stages, the basal keratinocytes layer exhibited also enhanced DNA damage response but in contrast to the one in HF progenitor was not associated with pro-inflammatory cytokines expression, but rather increased proliferation, lack of differentiation and immune response resembling psoriasiform dermatitis. Simultaneous *Nbn* and *Trp53* inactivation significantly exacerbated this phenotype, due to the lack of inhibition of pro-inflammatory cytokines secretion by Trp53. Altogether, we demonstrated novel functions of Nbn in HF maintenance and prevention of skin inflammation and we provide a mechanistic explanation that links cell intrinsic DNA maintenance with large scale morphological tissue alterations.

## INTRODUCTION

The Nijmegen Breakage Syndrome (NBS, OMIM, 251260) is an autosomal recessive disease characterized by microcephaly, congenital malformation, growth retardation, chromosomal instability, immunodeficiency, radiosensitivity, and tumor predisposition [1]. NBS is due to hypomorphic mutations of NBN. NBN forms with RAD50 and MRE11 the MRN complex. NBN alone or in the MRN complex is a protein essential for genomic stability involved in multiple biological pathways including cell cycle checkpoint activation upon DNA damage, DNA damage repair, DNA replication, telomere maintenance and chromatin remodeling [2]. Notably, the inactivation of *Nbn* is early embryonic lethal (E3.5), while the hypomorphic mutant mice are viable but recapitulate only partially the NBS phenotype [3]. The mechanisms responsible of the microcephaly, growth retardation, immunodeficiency and radiosensitivity, were extensively studied and identified [4]. Nevertheless, many orphans NBS phenotypes among them the skin defects remain poorly understood. Indeed, NBS patients are not only exhibiting predisposition to lymphoma or brain tumors but also to melanoma [5, 6]. *NBN* mutations/polymorphisms were also found in sporadic melanomas [7] and basal cell carcinoma [8]. In addition, NBS patients exhibited various skin abnormalities including abnormal pigmentation of the skin, sparse and thin hairs [4, 9], and less frequently cutaneous non caseating granulomas porokeratosis, and depigmentation [4, 10]. Altogether these findings suggest a key function of NBN in skin homeostasis. Recent studies highlighted the requirement of DNA damage response proteins including Atr [11, 12], Brca1 [13], and Prkdc [14] for skin development and HF progenitor maintenance. Notably, while *Trp53* inactivation rescues HF loss in Brca1-deficient mice [13], it actually accelerates and exacerbates the consequences of Atr deficiency on the skin [12].

To determine the importance of Nbn in skin homeostasis, we performed conditional post-natal Nbn inactivation in HF progenitors using *Egr2-Cre* mice [15, 16]. *Egr2-Cre* would allow the deletion of *Nbn* as early as P1 in HF progenitors and epidermis [17]. We found that Nbn deficiency in HF progenitors promoted increased DNA damage signaling, stimulating *Cdkn2a* (*p16^INK4A^*) up-regulation, Trp53 stabilization, secretion of cytokines such as Il6 and Il1b, growth arrest leading to progressive reduction of Krt15-positive (Krt15+) and Cd34-positive (Cd34+) cells and premature hair loss. At later stages, the basal keratinocytes layer exhibited also DNA Damage signaling with Cdkn2a and Trp53 response but these changes in contrast to those in HF progenitor were associated with the absence of pro-inflammatory cytokines expression, increased proliferation, lack of differentiation and immune response reminiscent of psoriasiform dermatitis. Simultaneous *Nbn* and *Trp53* inactivation significantly exacerbated this phenotype, mainly due to the lack of inhibition of pro-inflammatory cytokines secretion by Trp53.

## RESULTS

### Nbn deficiency leads to premature hair loss and thickening of epidermis

*Egr2-Cre* mediated deletion has been shown to begin as early as P1 in the skin [17]. The deletion is first affecting sparse and small areas in the back of the skin, then progress through the hair follicle and then the epidermis and in adult mice the vast majority of the skin tissues exhibit the deletion. As expected, when we performed immunohistochemistry on P18 *Nbn^Krox20-Cre^* skin, we observed an efficient depletion of Nbn first in the hair follicles (hair bulge and bulb) then progressively in the keratinocytes of the epidermis. Notably, the deletion is not completed and in the epidermis some cells remain Nbn-proficient (Suppl Figure 1A).

**Figure 1:**
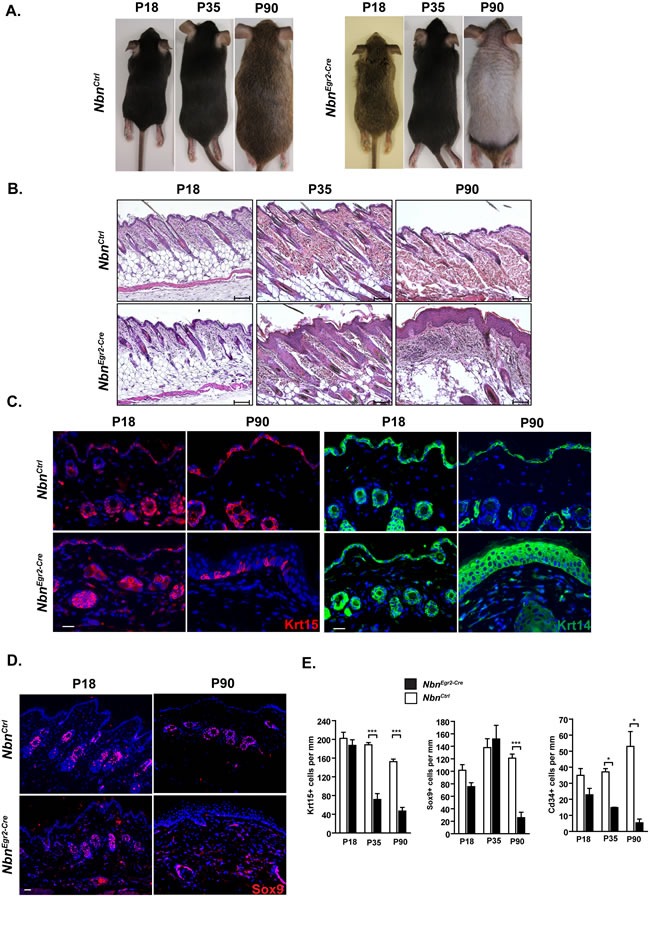
Nbn depletion leads to premature hair loss due to depletion of Krt15+ and Cd34+ HF progenitors **A.** Hair loss in *Nbn^Egr2-Cre^* skins from P35 to P90. Representative *Nbn^Ctrl^* and *Nbn^Egr2-Cre^* mice at different age, P18, P35 and P90. The hair loss can be observed from P35. **B.** Histological analysis of *Nbn^Egr2-Cre^* skin reveals progressive thickening of the epidermis and absence of HFs by P90. Scale bar 100 μm. **C.** Progressive depletion of Krt15+ HF progenitors and increase number of Krt14+ keratinocytes in *Nbn^Egr2-Cre^* skins. Scale bar 20 μm. **D.** Sox9 immunostaining in *Nbn^Ctrl^* and *Nbn^Egr2-Cre^* skins. **E.** Quantification of Krt15+, Sox9+ and Cd34+ cells in P18, P35 and P90 *Nbn^Egr2-Cre^* skins. The Krt15+, Sox9+ and Cd34+ cells were count per skin length (mm). For Krt15: *Nbn^Ctrl^* P18 (*N =* 4), *Nbn^Egr2-Cre^* P18 (*N =* 4), *Nbn^Ctrl^* P35 (*N =* 4), *Nbn^Egr2-Cre^* P35 (*N =* 4), *Nbn^Ctrl^* P90 (*N =* 2), *Nbn^Egr2-Cre^* P90 (*N =* 4). ***: *p* = 0.0003, **: *p* = 0.0018. For Sox9: *Nbn^Ctrl^* P18 (*N =* 2), *Nbn^Egr2-Cre^* P18 (*N =* 3), *Nbn^Ctrl^* P35 (*N =* 3), *Nbn^Egr2-Cre^* P35 (*N =* 3), *Nbn^Ctrl^* P90 (*N =* 3), *Nbn^Egr2-Cre^* P90 (*N =* 3). **: *p* = 0.0021. For Cd34: *Nbn^Ctrl^* P18 (*N =* 2), *Nbn^Egr2-Cre^* P18 (*N =* 3), *Nbn^Ctrl^* P35 (*N =* 3), *Nbn^Egr2-Cre^* P35 (*N =* 2), *Nbn^Ctrl^* P90 (*N =* 2), *Nbn^Egr2-Cre^* P90 (*N =* 4).*: *p* < 0.05.

We monitored *Nbn^Krox20-Cre^* mice over a period of three months. *Nbn^Krox20-Cre^* mice were indistinguishable from *Nbn^Ctrl^* mice in size and weight (Figure 1A, Suppl Figure 1B). Starting 35 days after birth (P35), *Nbn^Krox20-Cre^* mice exhibited reduction of hairs density (Figure 1A-1B). This phenotype continued to deteriorate until the complete lack of hairs by 90 days of age (P90) (Figure 1A-1B, Suppl Figure 1C). At P90, Histological analysis showed a complete hair loss in the *Nbn^Krox20-Cre^* epidermis but also a significant thickening of the keratinocytes layers (Figure 1B, Suppl Figure 1C). In order to identify the causes of these morphological changes, we first monitored the expression of cytokeratin 15 (Krt15), Cd34, and Sox9 that are HF (HF) stem cells markers [18] and cytokeratin 14 (Krt14) a marker of basal keratinocytes. In *Nbn^Krox20-Cre^* skins, the number of Krt15+ and Cd34+ cells progressively decreased from p18 to P35 while the number of Sox9-positive (Sox9+) cells was unaffected (Figure 1C-1E, Suppl Figure 1D-1F). At P90, only very few Krt15+ cells remained in the epidermis and nearly no Cd34+ and Sox9+ cells were observed (mainly due to the fact that at P90 *Nbn^Krox20-Cre^* skins exhibited a complete lack of hairs) (Figure 1C-1E, Suppl Figure 1E-1F). In contrast, the number of Krt14-positive (Krt14+) cells was dramatically increased in *Nbn^Krox20-Cre^* skins compared to *Nbn^Ctrl^* and was associated with thickening of the epidermis (Figure 1C). In summary, the data suggest that hair loss is caused by depletion of the Krt15+ and Cd34+ HF stem cells pool.

### HF depletion is associated with increased DNA damage signaling, growth arrest and secretory phenotype

**Figure 2:**
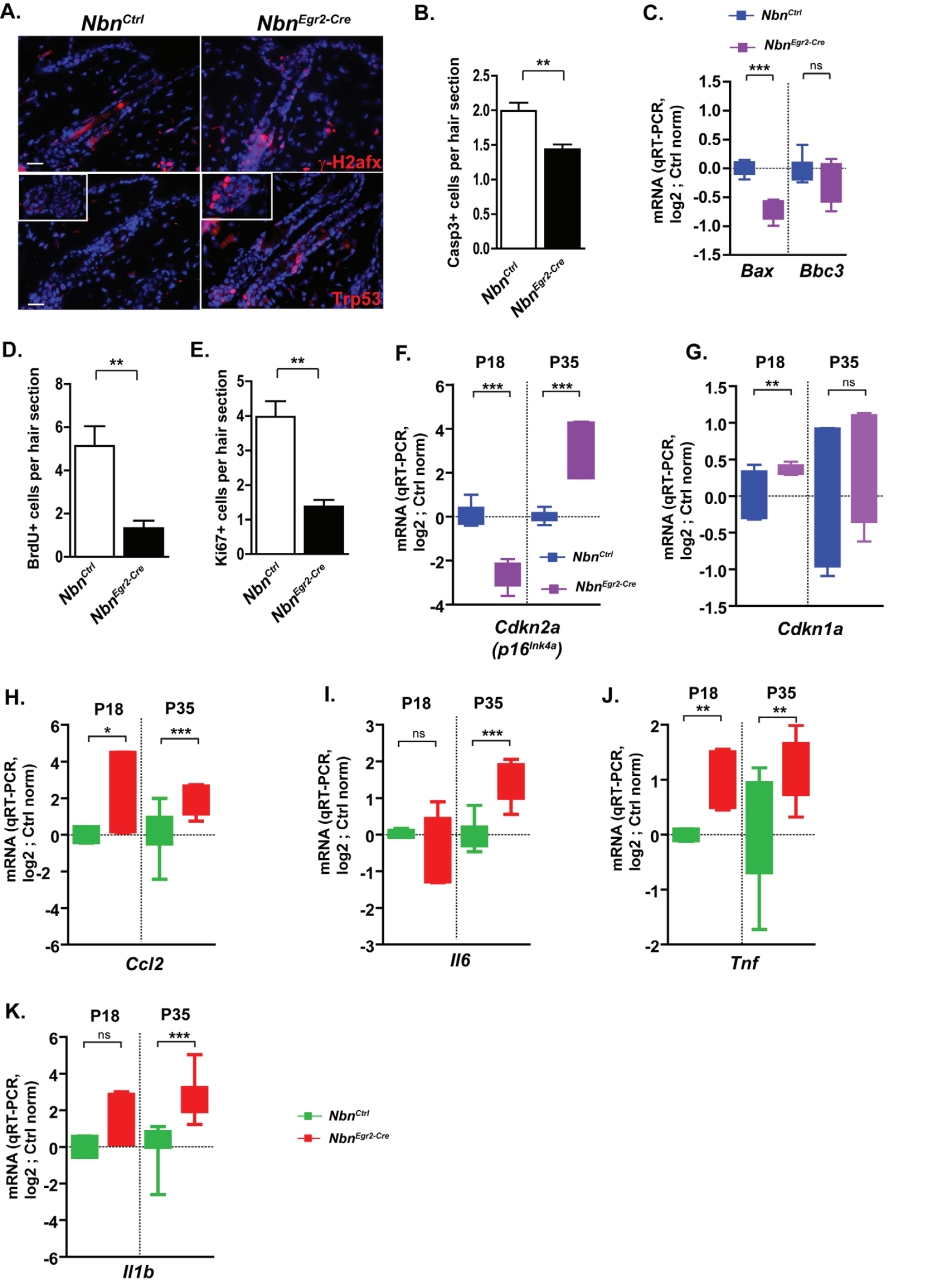
Nbn deficiency in HF progenitors triggers DNA damage response and secretion of pro-inflammatory cytokines **A.** Increase of γ-H2afx foci and Trp53 stabilization in *Nbn^Egr2-Cre^* HFs at P18. Scale bar 20 μm. **B.** Quantification of cleaved caspase 3 (Casp3+) in hair sections. *Nbn^Ctrl^* P18 (*N =* 3), *Nbn^Egr2-Cre^* P18 (*N =* 4). **C.** Real-time RT-PCR analysis of *Bax* and *Bbc3* (*Puma*) expression in *Nbn^Egr2-Cre^* P18 skins. *Nbn^Ctrl^* P18 (*N =* 4), *Nbn^Egr2-Cre^* P18 (*N =* 4). ***: *p* < 0.0001. **D.** Reduction of BrdU incorporation in *Nbn^Egr2-Cre^* HF sections. *Nbn^Ctrl^* P18 (*N =* 3), *Nbn^Egr2-Cre^* P18 (*N =* 4). *: *p* = 0.0108. **E.** Reduction of Ki67 positive (Ki67+) cells in *Nbn^Egr2-Cre^* HF sections. *Nbn^Ctrl^* P18 (*N =* 3), *Nbn^Egr2-Cre^* P18 (*N =* 4). *: *p* = 0.0108. Real-time RT-PCR analysis of *Cdkn2a* (*p16^INK4A^*) **F.** and *Cdkn1a* (*p21^Cip1/Waf1^*) **G.** expressions in *Nbn^Egr2-Cre^* P18 and P35 skins. *Nbn^Ctrl^* (*N =* 4), *Nbn^Egr2-Cre^* (*N =* 4). ***: *p* < 0.0001, **: *p* = 0.0077. Real-time RT-PCR analysis of pro-inflammatory cytokines: *Ccl2* **H.**, *Nbn^Ctrl^* P18 (*N =* 2), *Nbn^Egr2-Cre^* P18 (*N =* 3), *Nbn^Ctrl^* P35 (*N =* 6), *Nbn^Egr2-Cre^* P35 (*N =* 6); *Il6* **I.** *Nbn^Ctrl^* P18 (*N =* 2), *Nbn^Egr2-Cre^* P18 (*N =* 2), *Nbn^Ctrl^* P35 (*N =* 5), *Nbn^Egr2-Cre^* P35 (*N =* 5); *Tnf* **J.**, *Nbn^Ctrl^* P18 (*N =* 2), *Nbn^Egr2-Cre^* P18 (*N =* 3), *Nbn^Ctrl^* P35 (*N =* 5), *Nbn^Egr2-Cre^* P35 (*N =* 5) and Il1b **K.**, *Nbn^Ctrl^* P18 (*N =* 2), *Nbn^Egr2-Cre^* P18 (*N =* 3), *Nbn^Ctrl^* P35 (*N =* 6), *Nbn^Egr2-Cre^* P35 (*N =* 6). ***: *p* < 0.0001, **: *p* = 0.0085, *: *p* < 0.05.

Considering the central role of Nbn in DNA damage response signaling, we hypothesized that impaired DNA damage repair and progressive accumulation of DSBs leading to growth arrest or apoptosis could be at the origin of the observed phenotypes. Therefore, we analyzed γ-H2afx foci formation, cell proliferation and apoptosis. Interestingly, we observed a progressive increase of DSBs from 18 days after birth (P18) indicated by γ-H2afx foci accumulation in the hair bulge and hair germ (Figure 2A). We observed also Trp53 stabilization in HF sections as early as P18 (Figure 2A) suggesting an active DNA damage response that might lead to cell growth arrest or apoptosis. In addition, we were able to detect significant decreased apoptosis either by active Caspase-3 (ActCasp3) immunostaining, or qRT-PCR measurement of *Bax* from P18 to P90 (Figure 2B-2C, data not shown). In contrast, a significant reduction of BrdU and Ki67-positive cells in hairs follicle sections indicated cell growth arrest (Figure 2D-2E). The mRNA expression of *Cdkn2a*, one of the key markers of senescence was significantly increased between P18 and P35 (Figure 2F). Similarly, *Cdkn1a* (*p21^Cip1/Waf1^*) mRNA expression was also up-regulated as early as P18 (Figure 2G, Suppl Figure 1G).

After identifying HF DNA damage signaling-associated growth arrest as the cause for HF stem cells depletion; we were trying to find a biological link between the hair loss in one hand and the thickening of epidermis in the other hand. It was described recently a senescence-associated secretory phenotype (SASP) linked to chronic DNA damage-growth arrest which can simultaneously reinforce growth arrest of damage cells and stimulate neighboring cells to proliferate in paracrine fashion [19]. Therefore, we analyzed the mRNA expression of *Ccl2, Il6, Tnf* and *Il1b* by qRT-PCR. *Ccl2, Tnf* mRNA expression was induced as early as P18 (Figure 2H-2K) while *Il6* and *Il1b* up-regulation was delayed by P35 (Figure 2H-2K). The mRNA expression of these pro-inflammatory factors reached its maximum at P35 when the depletion of Krt15+ and Cd34+ HF stem cells intensified (Figure 2H-2K). At P90, their mRNA expression returned to basal levels (Suppl Figure 2C) suggesting that epidermal thickening at later stages was independent of this pro-inflammatory response. Altogether, the data indicate that Nbn-deficiency in HF stem cells led to γ-H2afx accumulation, growth arrest via the induction of *Trp53/Cdkn1a* and *Cdkn2a* and a pro-inflammatory response involving Ccl2, Il6, Tnf and Il1b up-regulation.

### Nbn-deficiency promotes a phenotype resembling psoriasiform dermatitis

**Figure 3:**
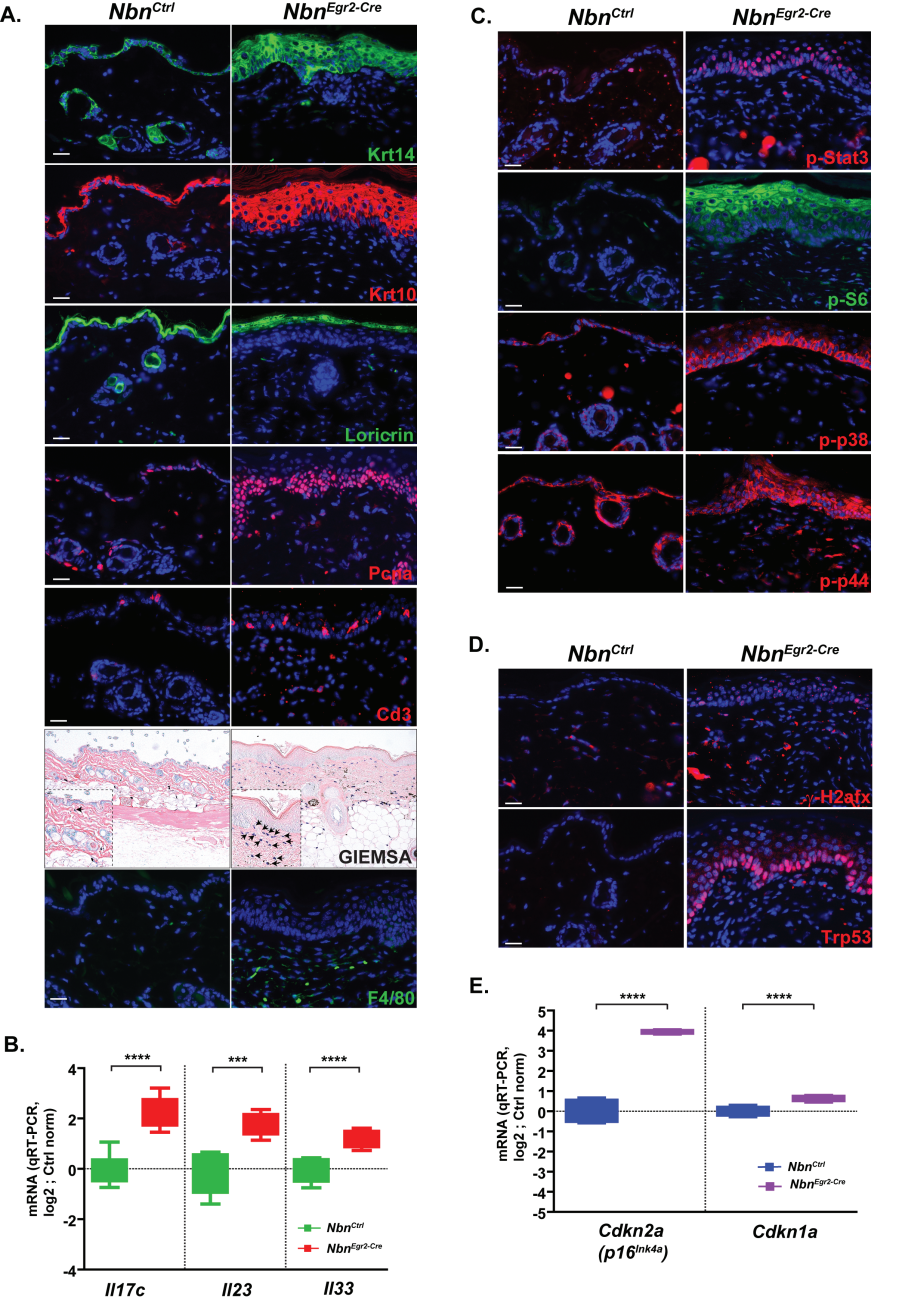
Nbn-deficiency promotes psoriasiform dermatitis **A.** Characterization of the psoriasiform dermatitis and inflammatory response using various cell type markers: Krt14, Krt10 and Loricrin; proliferation marker: Pcna (scale bar 20 μm). Immunohistochemistry of P90 *Nbn^Egr2-Cre^* skins indicates inflammatory response with T-cell marker (Cd3+, scale bar 20 μm), coloration of mast cells (blue) with GIEMSA (scale bar 40 μm) and macrophages (F4/80+). **B.** P90 *Nbn^Egr2-Cre^* skins highly expressed typical human psoriasiform dermatitis interleukins Il17c, Il23 and Il33. *Nbn^Ctrl^* (*N =* 4), *Nbn^Egr2-Cre^* (*N =* 4). **** : *p* < 0.0001, *** : *p* = 0.0002, ** : *p* = 0.0077. **C.** Up-regulation of p-Stat3, p-S6, p-p38 and p-p44 in P90 *Nbn^Egr2-Cre^* skins. **D.** γ-H2afx foci in keratinocytes trigger Trp53 stabilization and **E.** increased *Cdkn2a* (*p16^INK4A^*) and *Cdkn1a* (*p21^Cip1/Waf1^*) expression in P90 *Nbn^Egr2-Cre^* skins. *Nbn^Ctrl^* (*N =* 4), *Nbn^Egr2-Cre^* (*N =* 4). ****: *p* < 0.0001.

The histological analysis revealed that Nbn-deficiency in the skin led not only to HF loss but also to progressive thickening of the epidermis, acanthosis, parakeratosis and hyperkeratosis. To determine whether the phenotype was due to increased proliferation of the basal layer or lack of keratinocyte differentiation, we analyzed the structure of the epidermis using various keratinocytes markers. First, we monitored cytokeratin 10 (Krt10, in differentiation and differentiated keratinocyte marker) and Krt14 (Figure 3A). Interestingly, while in wild-type epidermis, Krt14+ cells composed at the maximum of two-three layers, in *Nbn^Krox20-Cre^* epidermis, Krt14+ cells often formed more than six layers at P90. Moreover, most of the Krt14+ cells were not quiescent and continued to proliferate as indicated by PCNA, Ki67 and phospho-H3 immunostaining (Figure 3A, data not shown). In addition, Krt10 staining revealed a thickening of the differentiated keratinocytes layer, and retention of the nuclei by the keratinocytes. These findings were also confirmed by the Loricrin staining. Indeed, while the Loricrin staining is restricted to the stratum corneum in control epidermis, many nucleated keratinocytes still expressed Loricrin in *Nbn^Krox20-Cre^* epidermis, indicating impaired enucleation and differentiation of the stratum corneum (Figure 3A, Suppl Figure 1H). Finally, similar to former studies [20], we observed a significant increased of melanocytes number in the skin at P90 (Suppl Figure 1I-1J). Altogether these data indicated that Nbn deficiency promotes cell proliferation and impairs normal differentiation of keratinocytes in the epidermis and favor melanocytes proliferation.

Epidermal thickening associated with acanthosis, parakeratosis and hyperkeratosis is a hallmark of psoriasiform dermatitis. Moreover, this disease is always associated with inflammation and immune response [21]. To analyze whether *Nbn^Krox20-Cre^* skin exhibit an immune response we monitored the infiltration of mast cells, Cd3-positive (Cd3+) T cells and macrophages (Figure 3A). As expected, we observed a progressive and significant increase of mast cell infiltration in the vicinity of the epidermis between P18 and P90 (Figure 3A, Suppl Figure 1K). Similarly, but with a significant delay, Cd3+ T cells and neutrophils progressively invade the lesions (Figure 3A, Suppl Figure 1L, data not shown). Since Egr2 is expressed in restricted stage of B- and T-Cell development [22] and deletion of *Nbn* could lead to B-and T-cells deficiency, we performed immunophenotyping of aged *Nbn^Krox20-Cre^* mice (Suppl Figure 2A-2B, data not shown). All the populations of B- and T- cells including Treg were present and confirmed the inflammation phenotype indicating that Nbn deletion in B- and T-cells using *Egr2-Cre* had no tremendous effects on B- and T- cells development. In addition, we observed enhanced mRNA expression of the interleukins *Il17c, Il23* and *Il33* which are key players in human psoriasiform dermatitis (Figure 3B). Finally, P90 *Nbn^Krox20-Cre^* epidermises exhibit increased phosphorylation/activation of Stat3 (p-Stat3), p38 (p-p38), p44 (p-p44) and S6 ribosomal protein (p-S6) which are also hallmarks of psoriasiform dermatitis (Figure 3C). Notably, the basal keratinocyte layer exhibits also increased γ -H2afx foci and p53 stabilization (Figure 3D), induction of *Cdkn2a* and *Cdkn1a* mRNA expression at P90 (Figure 3E), but no pro-inflammatory response as indicated by baseline mRNA expression levels of *Ccl2, Il6, Tnf* (Suppl Figure 2C).

In summary, Nbn-deficiency promotes psoriasiform dermatitis during ageing, exhibiting important accumulation of DNA damage in proliferative keratinocytes and induction of Trp53/Cdkn1a and Cdkn2a but lack of pro-inflammatory response *via* Tnf, Il6 and Il1b.

### *Trp53* inactivation exacerbates Nbn-deficient epidermal phenotype

**Figure 4:**
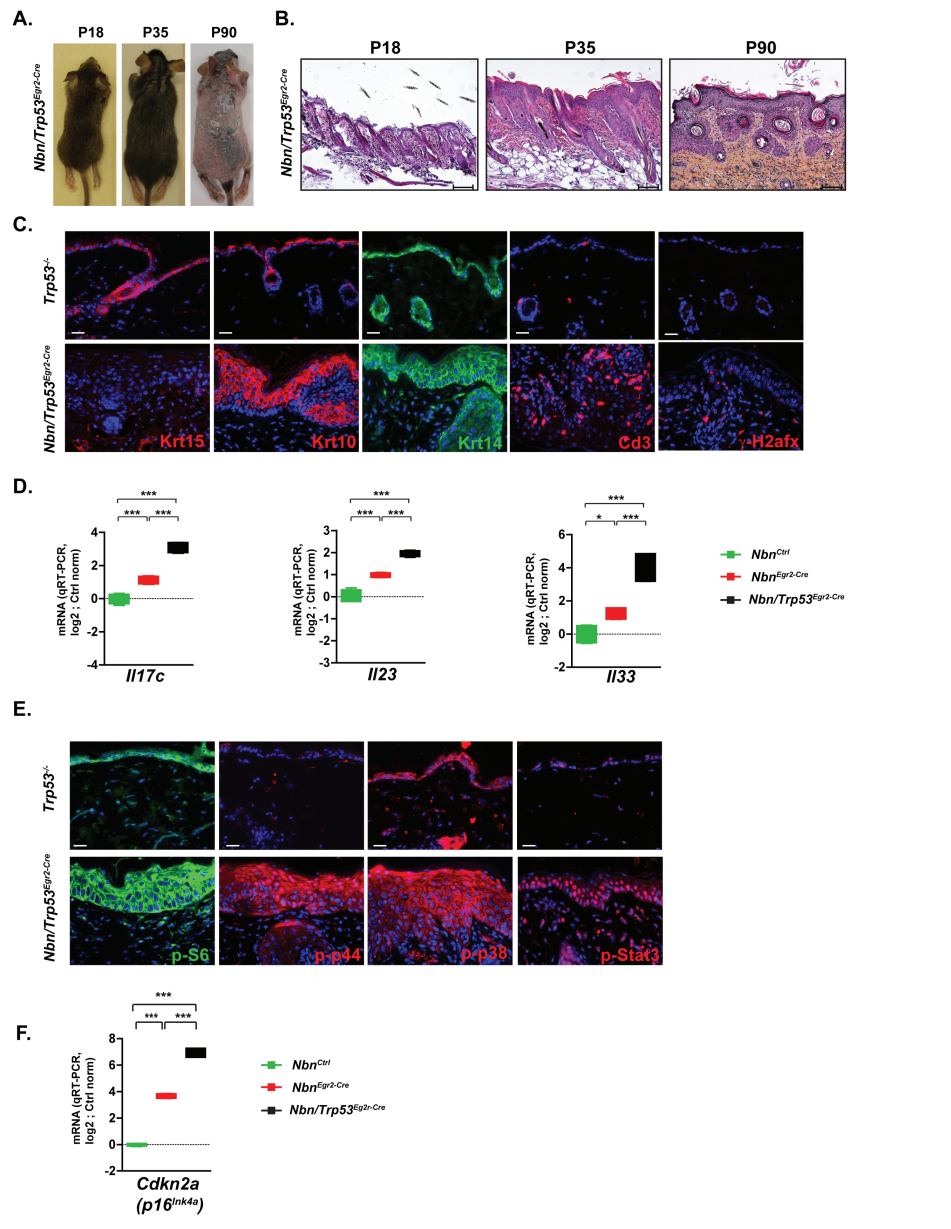
*Trp53* inactivation triggers worsening of *Nbn^Egr2-Cre^* phenotype **A.** The hair loss in *Nbn/Trp53^Egr2-Cre^* skins from P18 to P90 is similar to the *Nbn^Egr2-Cre^* ones **B.** Histological analysis of *Nbn/Trp53^Egr2-Cre^* skins reveals progressive aggravation of *Nbn^Egr2-Cre^* skins lesions by *Trp53* inactivation at P90. Scale bar 100 μm. **C.** Characterization of *Nbn*/*Trp53^Egr2-Cre^* skins using Krt15, Krt10, Krt14, Cd3+ and γ-H2afx staining. Remark the lack of Krt15+ cells, the ectopic localization of Krt10- and Krt14-positive cells and the high number of DSBs revealed by γ-H2afx foci accumulation in *Nbn/Trp53^Egr2-Cre^*. Scale bar 20 μm.**D.** Dramatic increase of *Il17c*, *Il23* and *Il33* expression in *Nbn*/*Trp53^Egr2-Cre^* skins (*Nbn^Ctrl^* (*N =* 2), *Nbn^Egr2-Cre^* (*N =* 2) and *Nbn/Trp53^Egr2-Cre^* (*N =* 2). **E.** Strong nuclear localization/activation of p-p44, p-p38 and p-Stat3 in *Nbn/Trp53^Egr2-Cre^* skins lesions. S6 phosphorylation occurs also in the basal keratinocytes layer. Scale bar 20 μm. **F.** Inactivation of *Trp53* is associated with increase of *Cdkn2a* expression in *Nbn/Trp53^Egr2-Cre^* skins. *Nbn^Ctrl^* (*N =* 2), *Nbn^Egr2-Cre^* (*N =* 2) and *Nbn/Trp53^Egr2-Cre^* (*N =* 2). ***: *p* < 0.001. Scale bar 20 μm.

It was shown that TP53 mitigated the effects of the senescence-growth arrest by indirectly suppressing the accumulation and propagation of DNA damage [19]. To analyze the role of Trp53 in the Nbn-deficient skin phenotype, it was decided to simultaneously inactivate Nbn and Trp53 in the HFs progenitors. Trp53-deficient skins were phenotypically/morphologically very similar to *Nbn^Ctrl^* skins. *Nbn^Krox20-Cre^* skins exhibited similar phenotype compared with the *Nbn^Krox20-Cre^* ones including premature hair loss (Figure 4A), epidermis thickening, acanthosis and parakeratosis (Figure 4B), increased γ-H2afx foci, lack of Krt15+ cells and immune cell infiltration (Figure 4C). However, some phenotypical modifications including hair invagination, ectopic localization of Krt10+ and Krt14+ keratinocytes and increased thickening of epidermis were exacerbated in *Nbn*/*^Trp53Egr2-Cre^* skins (Figure 4B-4C). This observation was confirmed at a molecular level first by a significant increase of the mRNA expression of psoriasiform dermatitis markers Il17c, Il23 and Il33 (Figure 4D). Interestingly, while general pro-inflammatory cytokines including *Il6, Il1b Tnf*, and *Ccl2* were expressed at their basal level in P90 *Nbn^Krox20-Cre^* skins, they were robustly induced in *Nbn/Trp53^Egr2-Cre^* skins (Suppl Figure 2C). Pro-inflammatory cytokines induction was associated with increased activation of p38, p44 and Stat3 pathways illustrated by increased nuclear localization of their phosphorylated forms (Figure 4E). Interestingly, while p-S6 was restricted to the upper layers and Krt10+ keratinocytes in *Nbn^Krox20-Cre^* epidermis, its expression in *Nbn/Trp53^Egr2-Cre^* spread to the basal layer (Figure 4E). These data indicated that in *Nbn^Krox20-Cre^* skins, *Trp53* inactivation dramatically worsens the Nbn-deficient phenotype by allowing the amplification of molecular pathways favoring impaired differentiation and proliferation thereby leading to the progression of psoriasiform dermatitis skins lesions towards eventually more advanced pre-neoplastic stages.

Considering the augmentation of mitotic activity and the enhanced pro-inflammatory phenotype, we expected that *Nbn/Trp53^Egr2-Cre^* skin lesions will finally evolve to tumour. However, for up to six months after birth, the skin lesions did not evolve indicating that pathways other than Trp53 prevent tumorigenesis. We could not detect any increased apoptosis between P18 and P90 (data not shown). However, the mRNA expression of *Cdkn2a* in *Nbn/Trp53^Egr2-Cre^* skins was significantly increased compared to *Nbn^Krox20-Cre^* skins (Figure 4F). These data suggest a strong compensatory activation of the Cdkn2a/Rb1 pathway in the absence of Trp53 to prevent tumorigenesis.

## DISCUSSION

In the past years, intensive research was performed to understand and modeling the etiology of psoriasiform dermatitis. Many mouse models were created but most of them failed to fully recapitulate the human psoriasiform dermatitis phenotype [23]. In this point, the *Nbn^Egr2-Cre^* mice presented in this study rapidly develop a complex skin phenotype with a 100% penetrance that reproduces the histological and molecular features of scaring alopecia and psoriasiform dermatitis including changes in cytokines expression and inflammatory response. To our knowledge, this is the first report demonstrating a link between DNA repair deficiency, the resulting chronic DNA damage response (DDR) and development of psoriasiform dermatitis. Indeed, we demonstrated that chronic DSBs and DDR on one hand trigger growth arrest and early pro-inflammatory signaling in HF stem cells leading to premature and permanent hair loss reminiscent of scaring alopecia [24, 25]. On the other hand, activation of intrinsic pro-inflammatory signaling pathways (Jak/Stat, p38 and p44), expression of both general (Il1b, Il6, Tnf and Ccl2) and more psoriasis-related (Il17c, Il23 and Il33) cytokines associated with a strong immune response were leading to a phenotype resembling psoriasiform dermatitis. However, due to the spatial and temporal specificity of the *Egr2* and *Nbn* expression, the model lacks for the most the ears, paws, tail and arthritic psoriatic lesions. In human, scaring alopecia can be caused by various factors including psoriasiform dermatitis [26, 27]. It was also described concomitant development of scaring alopecia and psoriasiform dermatitis upon treatments [28, 29]. However, in mice, similar to what we observe in our model, alopecia often precedes psoriasiform dermatitis [30, 31].

The majority of patients with DNA repair-deficiency syndromes such as NBS exhibit alopecia and a weak predisposition to skins tumours [4]. However, none of the DSBs repair-deficient patients or mice was shown to develop psoriasiform dermatitis at the exception of four LIG4 syndrome patients [32] and one NBS girl [33]. The presence of partially functional hypomorphic proteins and the severe immunodeficiency especially the lack of T-cells may explain the absence of pro-inflammatory response and psoriasiform dermatitis in DSBs-deficient associated human syndromes. T-cells and more particularly T_H_17 were proposed to play a strong role in the etiology and development of psoriasiform dermatitis [21]. However it was also shown that immune-compromised HIV patients exhibit the same rate of psoriasiform dermatitis as the general population [34] and that a complete inactivation of *Rag2* in a *JunB*/*JunC* mouse psoriasis model did not prevent the development of the disease [31, 35]. In addition, in our model, activated Cd3+ T-cells, macrophages and neutrophils were recruited late to the vicinity of the psoriatic skin lesions suggesting that they may be important for the maintenance of the psoriasiform dermatitis but not for its initiation. Only the mast cells recruitment coincided with the development of the psoriasiform dermatitis suggesting that mast cells may be required for its formation. Indeed, mast cells were previously demonstrated to play a critical role in the first stages of psoriasiform dermatitis in promoting keratinocyte proliferation and recruiting immune cells through secretion among others of IL6, and TNF [36, 37].

A variety of cytokines and chemokines have been shown to play a critical role in the etiology of psoriasiform dermatitis through a highly complex network of inhibition and stimulation. Among them the IL17 family and IL23 seem to be the most potent [38] and were also identified in our model. The IL17 family is composed of 6 members IL17A to F. To this day, the leukocyte-derived IL17A/F interleukins were the most studied and were described as the most important player in psoriasiform dermatitis [38]. However, new evidence indicates that IL17C secreted by epithelial cells and keratinocytes may be a key driver of the psoriasis [39]. Indeed, it is the most prominent interleukin in psoriasis plaques and its overexpression in keratinocytes is promoting psoriasiform skin inflammation [39]. Moreover, its functions overlap with those of IL17A [40] and its expression is stimulated both by IL1B and TNF [39] which preceded IL17C expression also in our model. Similarly, IL23 is produced by various cell types including dendritic cells, macrophages and epithelial cells [41] and is highly expressed in human psoriatic lesions [42]. Furthermore, it promotes epidermal hyperplasia in mouse [43, 44]. Interestingly, high IL6 and TNF expression were often associated with psoriasiform dermatitis [45, 46]. However, our results indicate that *Nbn^Egr2-Cre^* psoriasiform dermatitis maintenance does not require their continuous secretion. The HF stem cells may be the source of pro-inflammatory signaling *via* Il6 and Tnf in our model, based on the observations that DDR is active in those cells and their kinetics of depletion is correlated with the mRNA expression of these cytokines. However, it cannot be ruled out that basal keratinocytes could be the key drivers of inflammation through Il17c, as also suggested by recent findings in mouse and human psoriasiform dermatitis [39]. Furthermore, basal keratinocytes exhibit also DDR with no Il6 and Tnf induction in *Nbn^Egr2-Cre^* skins but with one in Nbn/Trp53-deficient skins showing that Trp53 is a strong regulator of the DDR-pro-inflammatory response and that basal keratinocytes at least in Nbn/Trp53-deficient skins might contribute to the exacerbation of the phenotype.

It was shown that senescence is a potent and irreversible growth suppressive mechanism that is activated upon various types of genotoxic or oncogenic stimuli [47] and that the two main regulatory pathways of senescence growth-arrest were TP53/CDKN1A and CDKN2A/RB1 [48, 49]. In addition, in a certain context, senescence-associated chronic DDR stimulate a senescence-associated secretory phenotype that implies the secretion of over 40 types of cytokines, chemokines, proteases and growth factors which have both beneficial and deleterious paracrine and autocrine effects [50]. The senescence growth-arrest is under the negative control of TP53 [51]. The data obtained with *Nbn^Egr2-Cre^* mice recapitulate in many aspects these findings with two exceptions. First, it was reported that *in vivo* Nbn depletion in neural progenitors triggers apoptosis rather than growth arrest [52] suggesting that the choice between growth arrest and apoptosis upon Nbn depletion is determined by the cell type and the stage of development and to a lesser extent by the degree and the type of DNA-damage. Second, it was reported that NBN positively regulated SASP and NBN depletion was associated with reduced IL6 secretion [53]. Surprisingly, we found a potent growth arrest associated secretion of Il6 and Il1b in *Nbn^Egr2-Cre^* mice. We hypothesized, that the different requirements of Nbn and the nature of the DNA lesions in these two models would explain these discrepancies. Indeed, Rodier et al, generated DNA damage using exogenous high dose ionizing radiation where Nbn was essential to fully activate DDR signaling and promote SASP response. In contrast, in our model, the origin of DNA damage is Nbn-deficiency itself and our current results and former ones [52, 54] already showed that it does not fully interfere with DDR.

In summary, we described for the first time that DNA damage associated pro-inflammatory phenotype can promote alopecia and trigger psoriasiform dermatitis development. This model resembles all the characteristics of the human disease. In addition it allows insight into the morphological and molecular disease progression and might therefore contribute to future mechanistic understanding and treatment. Therefore, psoriasiform dermatitis prevention measures and therapeutics development should take into consideration the contribution of DDR and secretory phenotype in the etiology and maintenance of the disease and how it could negatively affect skin homeostasis.

## MATERIALS AND METHODS

### Ethics statement

All animal care and procedures followed German legal regulations and were previously approved by the governmental review board of the state of Baden-Württemberg (Regierungspräsidium Karlsruhe-Abteilung 3-Landwirtschaft, Ländlicher Raum, Veterinär-und Lebensmittelwesen, experimental project. All the aspects of the mouse work were carried out following strict guidelines to insure careful, consistent and ethical handling of mice.

### Mice

The following mice were used *Nbn* floxed (B6;129-Nbs1^tm1Zqw^), *Trp53* knock-out (B6.129S2-Trp53^tm1Tyj/J^), *Trp53* floxed (FVB.129-Trp53^tm1Brn^). *Egr2-Cre* mice (B6; 129- Egr2^tm2(Cre)Pch^) were a generous gift form P. Charnay. The *Nbn* construct and the deletion was described in details in Demuth et al [55], and Frappart et al [54] and the pattern of deletion by *Egr2-Cre* was well described in Voiculescu et al [16] and Gambardella et al. [15].

### Histology and Immunohistochemistry

Skins were collected in 4% (w/v) phosphate-buffered saline (PBS)-buffered paraformaldehyde (PFA) for 24 hours at 4¼C. 5 μm paraffin sections were performed. For cryosections, the tissues were cryopreserved in sucrose solution 25% and 10 μm cryosections were performed. The following antibodies required 40 min citrate buffer antigen retrieval at 95°C: anti-active caspase3 (BD bioscience, 559565), anti-BrdU (NovusBio, NB500-169), anti-ser139 γ-H2AFX (Millipore, 05-636), anti-ser235/236-S6 Ribosomal Protein (Cell Signaling, 2211), anti-Thr180/Tyr182-p38 MAPK (D3F9, Cell Signaling, 4511) anti-Thr202/Tyr204-p44/p42 MAPK (D13.14.4E, Cell Signaling, 4370), anti-Tyr705-Stat3 (D3A7, Cell Signaling, 9145), anti-Ki67 (SP6, Thermo Scientific, RM9106S), anti-PCNA (PC10, Santa Cruz Biotechnology, sc-56), anti-Trp53 (CM5, Novocastra, NCL-p53-CM5p), anti-cytokeratin10 (Covance, PRB-159P), anti-cytokeratin14 (Covance, PRB-155P), anti-cytokeratin 15 (Abcam, ab80522), anti-neutrophils (Acris, CL050RX), anti-CD3 (SP7, Rockland immunochemicals, 900-C01-B39), anti-macrophage F4/80 (Acris, BM4007B), anti-CDKN1A (p21^Cip1/Waf1^) (F-5, Santa Cruz Biotechnology, sc-6246), anti-Sox9 (Millipore, AB5535). The following antibody required 10 min EDTA buffer antigen retrieval at 95°C, anti-CD34 (RAM34, BD bioscience, 553731).

Immunoreactivity was visualized using secondary donkey antibodies conjugated with biotin (Jackson Immunoresearch) followed by the incubation with Cy3 or FITC-conjugated Streptavidin (Sigma-Aldrich, GEPA43001). Nuclei were counterstained with Hoechst 33342 (1:10000, Life Technologies) and mounted in ProLong Gold Antifade reagent (Life Technologies). For the *in vivo* proliferation study, mice were injected intraperitoneally with 50 μg/g body weight with BrdU (Sigma-Aldrich, B9285). Skins were collected 1 hour after injection.

Nbn immunohistochemistry was performed using Ventana BenchMark^®^ XT instrument (Ventana Medical Systems) using Ventana reagents (OptiView HQ Universal Linker, OptiView HRP Multimer, OptiView Amplifier and OptiView Amplifier Multimer, UltraWash, counterstaining, UltraView Universal DAB Detection Kit) and following manufacturer's instructions. The following antibodies were used: anti-NBN (Sigma-Aldrich, HPA001429), anti-NBN (Y112, Millipore, 04-236)

The images were captured with Zeiss Axiovert fluorescence microscope.

### mRNA extraction and expression analysis

For mRNA analysis, skin samples were harvested, snap-frozen in liquid nitrogen and stored at −80°C. The tissue was then subjected to RNA isolation using the RNeasy Mini kit (Qiagen) according to the manufacturer's instructions. The total RNA yield and quality were determined using NanoDrop (Thermo Fisher Scientific) and Agilent Bioanalyzer (Agilent Technologies). 1 μg of total RNA were reverse transcribed using the High Capacity RNA-to-cDNA^™^ kit (Life Technologies) following the manufacturer's instructions. cDNA was mixed with gene-specific TaqMan^®^ Primer and Probes (Life Technologies) and TaqMan^®^ Universal Master Mix (Life Technologies). Quantitative RT-PCR (qRT-PCR) was performed using a 7900HT Fast Real-Time PCR System (Life Technologies). For analysis, raw Ct values were normalized against average expression from housekeeping genes (*Gapdh*, *Ubc*, *ActB*) within the same cDNA sample and against target expression in cDNA samples from matched wild-type littermates (ddCt method). List of mouse TaqMan^®^ Primer and Probes: *Gapdh* (ABI-Mm99999915_g1), *Ubc* (ABI-Mm01198158_m1), *Actb* (ABI-Mm00607939_s1), *Il1b* (ABI-Mm00434228_m1), *Il6* (ABI-Mm99999064_m1), *Tnf* (ABI-Mm00443258_m1), *Ccl2* (ABI-Mm00441242_m1), *Cdkn1a* (*p21^Cip1/Waf1^*) (ABI-Mm01303209_m1), *Cdkn2a* (*p16^Iink4a^*) (ABI-Mm00494449_m1), *Bax* (ABI-Mm00432051_m1), *Bbc3* (*Puma*) (ABI-Mm00519268_m1), *Il17c* (ABI-Mm00521397_m1), *Il23* (ABI-Mm01160011_g1), *Il33* (ABI-Mm00505403_m1), *Il10* (ABI-Mm00439614_m1).

### Immunophenotyping of Nbn^Egr2-Cre^ mice

The spleen and the inguinal, axial, brachial lymph nodes from *Nbn^Ctrl^* and *Nbn^Egr2-Cre^* mice were collected and a single cell suspension was obtained by mashing and filtering the tissues followed by ammonium chloride—potassium bicarbonate erythrocyte lysis. Intracellular staining of cells was performed using the intracellular Foxp3 staining buffer set (eBioscience) according to the manufacturer's protocol. Cells were analyzed using the BD Bioscience Canto II. Treestar FlowJo was used for the analysis of flow cytometry data. The following antibodies were used: anti-CD4 (BD Biosciences, 563790), anti-CD8 (Biolegend, 100744), anti-CD25 (Biolegend, 102008), Foxp3 (eBiosciences, 17-5773-82), CD357 (GITR) (Biolegend, 126308), CTLA-4 (Biolegend, 106305), Helios (Biolegend, 137206), CD304 (NRP1) (MBL, M169-A48).

### Statistical analysis

Quantification analysis of immunopositive cells was performed on paraffin sections either by counting the number of positive cells per mm of skin surface (mm) measured on the paraffin sections or per hair sections in at least 5 representative random sections and mean values were calculated and statistically analyzed. These data were collected from at least 3 different animals per each group. *T*-Test and One way ANOVA followed by post hoc Newman-Keuls analysis were performed using Prism (v5.0, Graphpad). Differences were considered significant when *P* value was < 0.05.

## SUPPLEMENTARY MATERIAL FIGURES


