# Supplementary material for: Epidermal Nbn deletion causes premature hair loss and a phenotype resembling psoriasiform dermatitis
Source: Oncotarget. 2016 Mar 30;7(17):23006–18. doi: 10.18632/oncotarget.8470 (PMC5029606; doi:10.18632/oncotarget.8470)
Supplement: Supplementary file 1 [file oncotarget-07-23006-s001.pdf]

Epidermal *Nbn* deletion causes premature hair loss and a phenotype resembling psoriasiform dermatitis

Supplementary Material

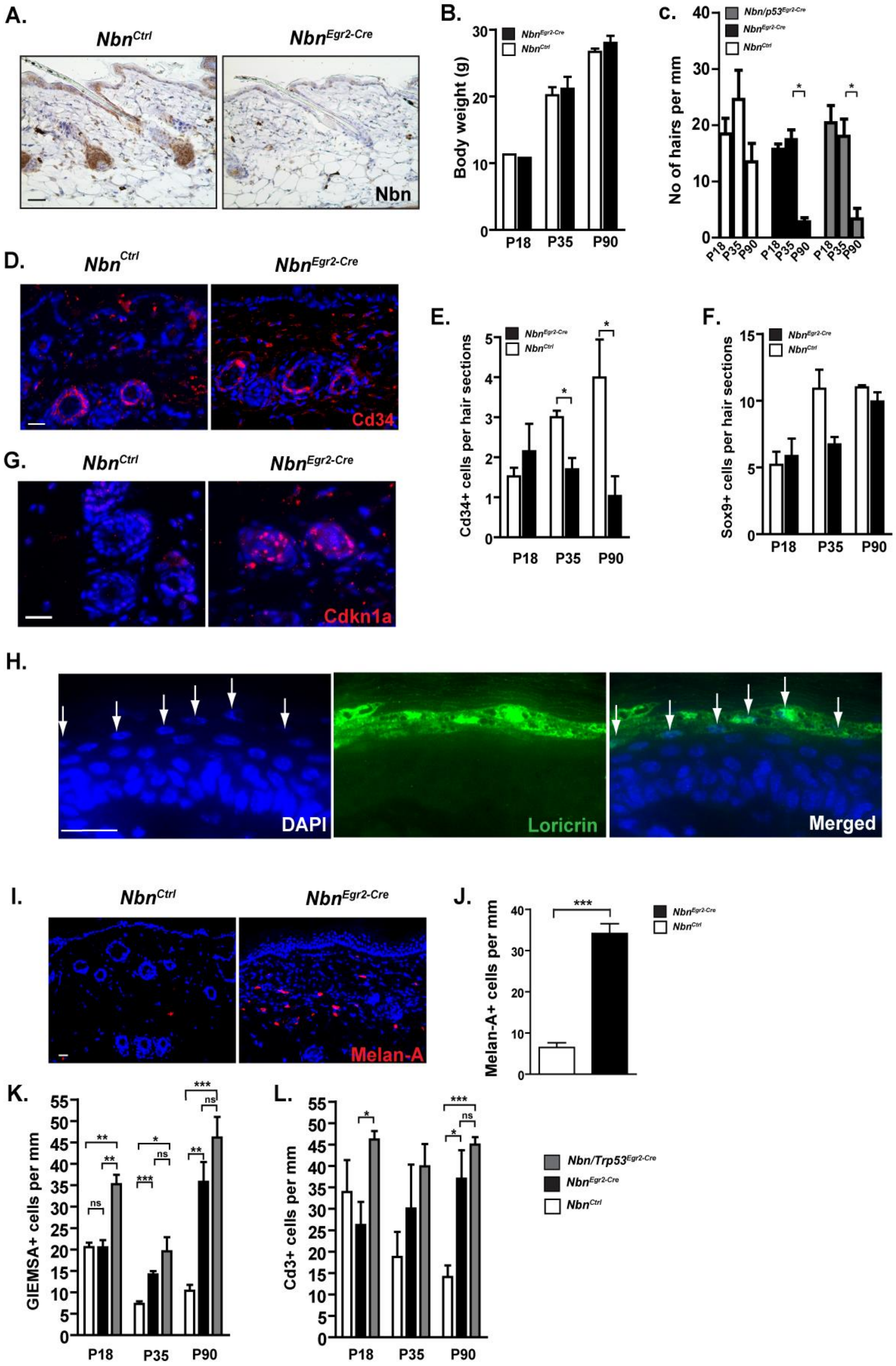

Supplementary Figure 1: (**A**) Nbn Immunohistochemistry shows Nbn expression in HF bulk and keratinocytes at P18. Remarks the dramatic reduction of Nbn expression in $Nbn^{Egr2\text{-}Cre}$ skins. (**B**) Body weight of Nbn^{Ctrl} and $Nbn^{Egr2\text{-}Cre}$ mice at P18, P35 and P90. No obvious weight differences were observed. Nbn^{Ctrl}: P18 (N=1), P35 (N=5), P90 (N=3). $Nbn^{Egr2\text{-}Cre}$: P18 (N=1), P35 (N=2), P90 (N=5). (**C**) Quantification of HFs in P18, P35 and P90 $Nbn^{Egr2\text{-}Cre}$ skins. The HFs were count in section of skin and measure per skin length (mm). At P18, Nbn^{Ctrl} (N=9), $Nbn^{Egr2\text{-}Cre}$ (N=6), $Nbn/Trp53^{Egr2\text{-}Cre}$ (N=4); at P35 Nbn^{Ctrl} (N=7), $Nbn^{Egr2\text{-}Cre}$ (N=5), $Nbn/Trp53^{Egr2\text{-}Cre}$ (N=3); at P90 Nbn^{Ctrl} (N=6), $Nbn^{Egr2\text{-}Cre}$ (N=7), $Nbn/Trp53^{Egr2\text{-}Cre}$ (N=4). ***: p=0.0002. (**D**) Representative Cd34 immunostaining in P18 Nbn^{Ctrl} and $Nbn^{Egr2\text{-}Cre}$ skins. Scale bar 20 µm. (**E**) Quantification of Cd34+ cells in hair sections. A significant difference was observed between Nbn^{Ctrl} and $Nbn^{Egr2\text{-}Cre}$ HFs as early as P35. Nbn^{Ctrl} at P18 (N=2), at P35 (N=2), at P90 (N=3). $Nbn^{Egr2\text{-}Cre}$ at P18 (N=3), at P35 (N=2), at P90 (N=3). * p < 0.05. (**F**) Quantification of Sox9+ cells in hair sections. Nbn^{Ctrl} at P18 (N=2), at P35 (N=3), at P90 (N=3). $Nbn^{Egr2\text{-}Cre}$ at P18 (N=3), at P35 (N=3), at P90 (N=3). No significant difference was observed between Nbn^{Ctrl} and $Nbn^{Egr2\text{-}Cre}$ HFs. (**G**) Cdkn1a ($p21^{Cip1/Waf1}$) positive immunostaining in P18 $Nbn^{Egr2\text{-}Cre}$ HF sections. (**H**) Loricrin staining of P90 $Nbn^{Egr2\text{-}Cre}$ indicating the retention of nucleus in Loricrin-positive cells. Scale bar 20 µm. (**I**) Representative melanocytes immunostaining using Melan-A marker. Scale bar 20 µm. (**J**) Quantification of Melan-A positive (Melan-A+) cells per mm of skin length in Nbn^{Ctrl} (N=4) and $Nbn^{Egr2\text{-}Cre}$ (N=4). A significant increase of the number of melanocytes was observed in $Nbn^{Egr2\text{-}Cre}$ skins. *** : p<0.001. Quantification of GIEMSA+ (**K**) and Cd3+ (**L**) cells per surface skin mm in Nbn^{Ctrl}, $Nbn^{Egr2\text{-}Cre}$ and $Nbn/Trp53^{Egr2\text{-}Cre}$ skins. At P18, Nbn^{Ctrl} (N=3), $Nbn^{Egr2\text{-}Cre}$ (N=4), $Nbn/Trp53^{Egr2\text{-}Cre}$ (N=3); at P35 Nbn^{Ctrl} (N=4), $Nbn^{Egr2\text{-}Cre}$ (N=4), $Nbn/Trp53^{Egr2\text{-}Cre}$ (N=3); at P90 Nbn^{Ctrl} (N=5), $Nbn^{Egr2\text{-}Cre}$ (N=5), $Nbn/Trp53^{Egr2\text{-}Cre}$ (N=3). *: p<0.05, **: p<0.01, ***: p<0.001, ns: no significant.

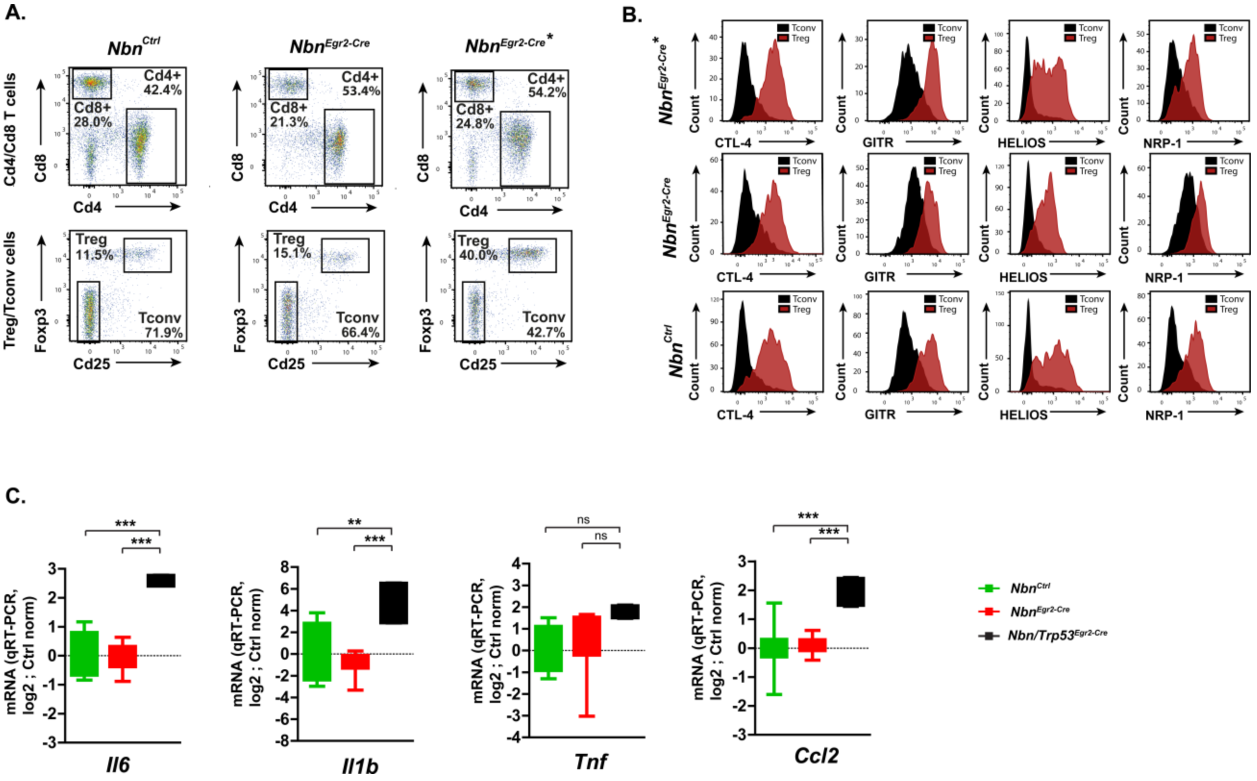

Supplementary Figure 2: (**A**) Immunophenotyping of *Nbn*$^{Egr2-Cre}$ mouse. Cd4 positive (Cd4+) and Cd8 positive (Cd8+), Foxp3 positive (Foxp3+; regulatory T cells) T cell population in 5 months old *Nbn*^{Ctrl} and *Nbn*$^{Egr2-Cre}$ mice (* indicates that this mouse had a splenomegaly and severe lymphadenopathy). The number written below the indicated box are the percentage of the respective subpopulation of T cells. (**B**) Expression pattern of indicated marker are shown for regulatory T cells (Tregs; defined as Cd4+Foxp3+Cd25+) and conventional T cells (Tconv). **(C**) Persistence of inflammatory response indicated by *Il6*, *Il1b*, *Tnf*, *Ccl2* expression in *Nbn/Trp53*$^{Egr2-Cre}$ at P90. *Nbn*^{Ctrl} (N=4), *Nbn*$^{Egr2-Cre}$ (N=4), *Nbn/Trp53*$^{Egr2-Cre}$ (N=2).
